# National Clinical Database (NCD) in Japan: Clinical and social significance

**DOI:** 10.1002/ags3.12282

**Published:** 2019-08-12

**Authors:** Hideo Baba

**Affiliations:** ^1^ Department of Gastroenterological Surgery Graduate School of Medical Sciences Kumamoto University Kumamoto Japan

Gastroenterological (GE) cancer is the leading cause of cancer‐related death, with the highest mortality rate worldwide. Although the recent development of multidisciplinary treatments has improved the prognosis of GE cancer, surgical treatment remains the mainstay of therapy. However, the clinical significance of extended surgery for advanced GE cancer has been contradicted by large‐scale clinical trials. It is well known that invasive surgical procedures or postoperative complications can lead to adverse effects not only on short‐term but also on long‐term outcomes for GE cancer. Many hospitals and surgeons have trouble tracking surgical complications and may lack the data necessary to analyze and take appropriate steps to correct problems. We cannot improve our surgical quality if we do not properly measure it, and so it is essential to establish a nationally validated, risk‐adjusted, outcomes‐based system to measure and improve the quality of surgical care.

In Japan, the National Clinical Database (NCD) was founded in 2010 as the parent body of the database system linked to the board certification system.^1^ The NCD project, which began recordkeeping in January 2011, contains records of ≥95% of the operations carried out by regular surgeons in Japan. Almost 5000 facilities have enrolled, and over 9 690 000 cases had already been registered from January 2011 to the end of December 2017. Various risk stratification studies based on data from the NCD database have been reported for the various types of GE surgery for Japanese patients. Furthermore, these risk‐scoring systems use a risk calculator that is available on the NCD website for physicians in clinical practice. Most recently, the data accuracy of the cases of GE surgery registered in the NCD was reported to be high, with a high overall concordance rate.^2^

Two reports regarding GE surgery using NCD data appear in the present issue of *Annals of Gastroenterological Surgery*. Hata et al show real‐world data in Japan regarding the frequency of and risk factors for venous thromboembolism (VTE) after GE surgery in 516 217 patients. Deep vein thrombosis was observed in 0.3% and pulmonary embolism in 0.2% of these patients. Esophagectomy, pancreatoduodenectomy and hepatectomy were identified as risk factors for VTE. Based on this finding, we should reconsider the use of prophylaxis anticoagulant medication for these procedures. Haga et al constructed a prediction model to estimate grade‐specific postoperative morbidity in gastrectomy using NCD data. This model can predict more precisely severe‐grade complications, such as grade ≥4 or grade 5, with good discriminative and calibration power. We believe that these data provide valuable information for assessing average risk in GE surgery, contributing to informing patients and their families of the risks associated with each GE surgical procedure.

In addition to the above clinical utility of the NCD, it is likely that the NCD system will have several promising applications in clinical practice. First, although information on long‐term outcomes, such as recurrence‐free survival and overall survival, has not been registered in the NCD, we have been able to register prognostic data for gastric cancer through the NCD system since September 2018. We hope that the linkage between short‐ and long‐term information can result in further establishment of clinical evidence from Japan. Second, Konno et al investigated the association between board‐certified surgeons in GE surgery and the surgical outcomes of GE surgery using NCD data.^3^ They demonstrated that board‐certified GE surgeons contribute to favorable outcomes of GE surgery in Japan. These data show that the board‐certified GE surgeon system in Japan has high validity, which can help us to improve the quality of board‐certified GE surgeons and to establish a new board‐certified system. Finally, we can identify problem areas and improvements for surgical treatment by fair, in‐depth and insightful analysis of NCD data. Although the Japanese government has recently executed an action plan for reform of working practices, we need to retain surgical quality as well as attempt to remedy the situation. We hope that analyses of nationwide data will provide some highly promising approaches for the reform of working practices of GE surgeons.

## DISCLOSURE

Conflicts of Interest: Author declares no conflicts of interest for this article.
